# Determination of ^137^Cs and ^90^Sr in wood fuels and their ashes available in Austria

**DOI:** 10.1093/rpd/ncaf036

**Published:** 2025-08-28

**Authors:** Viktoria Herzner, Christian Katzlberger, Christoph Pfeifer, Franz Josef Maringer, Martin Weigl-Kuska

**Affiliations:** Department for Technical Radiation Protection, Austrian Agency for Health and Food Safety, Spargelfeldstraße 191, 1220 Vienna, Austria; Department for Technical Radiation Protection, Austrian Agency for Health and Food Safety, Spargelfeldstraße 191, 1220 Vienna, Austria; Department of Natural Sciences and Sustainable Resources, Institute of Chemical and Energy Engineering, BOKU University, Muthgasse 107, 1190 Vienna, Austria; Department of Ecosystem Management, Climate and Biodiversity, Institute of Soil Research, BOKU University, Peter-Jordan-Straße 82, 1190 Vienna, Austria; Atominstitut, Unit Radiation Physics, TU Wien, Stadionallee 2, 1020 Vienna, Austria; Unit Circular economy, Holzforschung Austria, Franz-Grill-Straße 7, 1030 Vienna, Austria

## Abstract

This study assessed the radiological risk of wood fuels and their ashes in Austria, including wood chips, logs, pellets, and briquettes. Commercially purchased wood fuels are often of unknown origin and may have been imported. ^137^Cs activity concentrations were measured in wood fuels (69 samples) and their ashes (27 samples) using gamma-ray spectrometers with high-purity germanium detectors. ^90^Sr analyses were performed on 12 ash samples after chemical separation using PerkinElmer 1220 Quantulus™ liquid scintillation counters. Results showed ^137^Cs activity concentrations ranging from 0.327 to 8.36 Bq kg^−1^ in wood fuels (average 2.1 Bq kg^−1^) and from 11.80 to 867 Bq kg^−1^ in ashes (average 310 Bq kg^−1^). The ^90^Sr activity concentrations in ashes ranged from 363 to 1200 Bq kg^−1^ (average 655 Bq kg^−1^). Summarizing, this study suggests that wood fuels currently available in Austria do not pose a significant radiological risk from their ashes, negating the need for import regulations.

## Introduction

If wood is produced in a sustainable way (e.g. proved via certification such as PEFC (= Programme for the Endorsement of Forest Certification) [1]), its bioenergy (= biomass for energy) can under certain circumstances reduce greenhouse gas emissions [2]. Since 2018, the Austrian government has implemented subsidy programs to support the transition from fossil fuel-based space heating to sustainable heating systems (e.g., wood heating) [3, 4].

There are specially designed and optimized combustion systems for different wood fuels (wood chips, split logs, wood pellets, and wood briquettes). In recent years, an increasing number of households in Austria have adopted pellet boilers. There are more pellet boilers nationwide than other biomass boilers [5]. In Austria, pellets for the non-industrial market are produced only with by-products from the sawmill industry and without bark. There are more than 40 different pellet production sites in Austria and 11 more will be built by 2024 [6].

When heated, the organic part of wood burns, while the mineral, non-combustible part remains as ash. Plant ashes from biomass firing can be valuable secondary raw materials. However, their possible uses on agricultural and forestry land should be considered with care.

The Chornobyl fallout and nuclear weapon tests contaminated Europe, inter alia, with ^134^Cs, ^137^Cs, ^89^Sr, and ^90^Sr. These radionuclides are absorbed by the vegetation in the contaminated areas [7]. Due to their half-life of 30.08 and 28.90 years [8], 37 years later ^137^Cs and ^90^Sr can still be detected in the vegetation and wild animals. If wood from these regions is used for heating, radioactive cesium and strontium may accumulate in the ashes.

The uptake of ^137^Cs from soil into stemwood depends on the tree species and the site specifics like soil type and soil moisture [9]. The activity concentration of ^137^Cs varies considerably within a tree. For example, the radionuclides accumulate by deposition mainly in the bark, but the bark is easily decontaminated again by rainwater and by debarking [9]. The activity concentration of ^137^Cs is higher in the bark than in the heartwood [10]. Due to their bioaccumulation, the concentration of mineral salts and heavy metals is usually higher in bark [11].

^137^Cs is mobile within a tree and accumulates mainly in the growing parts of the tree, such as the leaves, needles, branches, and outer layers [12, 13]. The artificial radionuclide ^137^Cs follows qualitatively, but not quantitatively, the metabolic pathway of the natural potassium [14, 15]. In heartwood, the concentration of ^137^Cs is generally lower than in the growth zones [14, 15].

For chips and logs, the whole log (with bark) is usually used as wood fuel. It is not possible to determine exactly which parts of the tree (excl. the bark) are used for the production of pellets and briquettes.

The problem of accumulation of radionuclides such as ^137^Cs in the ash caught media attention in 2009 when Italy seized 10 000 tons of imported wood pellets from Lithuania on suspicion of ^137^Cs contamination [16].

There is no uniform regulation within the European Union (EU) for ^137^Cs activity concentration in wood and its combustion residues. In general, a reporting value for ^134^Cs and ^137^Cs of 10 000 Bq kg^−1^ applies in the EU [17]. Table 1 shows various regulations on activity concentrations of ^137^Cs and ^90^Sr, covering both EU and non-EU countries.

**Table 1:** Overview of various regulations on activity concentrations of ^137^Cs and ^90^Sr in wood, for both EU and non-EU countries.

Country	Restriction	Maximum allowed specific radioactivity of the radionuclide in wood (unless otherwise specified)
Belgium^*^ [18]	Firewood	^137^Cs 1000 Bq kg^−1^
Latvia^*^ [19]	Wood is imported into Latvia for use as biomass fuel	^137^Cs 10 Bq kg^−1^ in dry wood
	May be used in agriculture and forestry in accordance with the laws and regulations in the relevant sector	^137^Cs in wood ash ≤1000 Bq kg^−1^ in dry ash
	May be disposed of in a landfill site for municipal waste or used for the formation of coverings in a landfill site for municipal or hazardous waste	^137^Cs in wood ash >1000 Bq kg^−1^ and < 10 000 Bq kg^−1^ in dry ash
	Ash is managed in accordance with the laws and regulations regarding the requirements for activities with radioactive waste and materials related thereto	^137^Cs in wood ash ≥10 000 Bq kg^−1^ in dry ash
Sweden^*^ [20] (for combustion plants producing ≥100 t of ash per year)	Contaminated ash	^137^Cs in wood ash >1000 Bq kg^−1^ in dry ash
	Sum formula <1 Recycled: on forest land (not on lichen land in the reindeer husbandry area) road or fill material outdoors (dose rate < 0.5 μSv h^−1^at 1 m above paved surface)	^137^Cs in wood ash <10 000 Bq kg^−1^ in dry ash
	Disposed at non-hazardous waste or hazardous waste landfill	^137^Cs in wood ash ≥10 000 Bq kg^−1^ in dry ash
Belarus [21]	Fuel wood	^137^Cs 30 Bq kg^−1^
Japan [22]	Firewood for cooking and heating	radioactive cesium 40 Bq kg^−1^ (dry weight)
Ukraine [23]	Firewood, fuel bundles	^137^Cs 600 Bq kg^−1^ ^90^Sr 60 Bq kg^−1^
Russia [24]	Logs for the preparation of axes, handles, rakes, architraves, parts of tools and agricultural implements and other wood products used for industrial purposes and outside residential premises	^137^Cs 3100 Bq kg^−1^ ^90^Sr 2300 Bq kg^−1^
	Logs for the manufacture of furniture, parquet, caskets and other products used in everyday life, residential and public premises; resonance log for making musical instruments	^137^Cs 2200 Bq kg^−1^; ^90^Sr 520 Bq kg^−1^
	Fuel wood	^137^Cs 1400 Bq kg^−1^; ^90^Sr 370 Bq kg^−1^
	Wood for housing construction	^137^Cs 3700 Bq kg^−1^; ^90^Sr 5200 Bq kg^−1^

^*^Member state of the European Union.

In Austria, practices with wood ashes are currently not explicitly covered by the regime of the Federal Act on measures to protect against the dangers arising from ionizing radiation (Radiation Protection Act 2020—StrSchG 2020). This study evaluates the radiological risk of wood ashes to the public.

The activity concentrations in wood itself do not pose a health risk for the population and are harmless from the point of view of radiation protection. However, the accumulation of radionuclides in the resulting wood ash varies greatly. Depending on the ^137^Cs and ^90^Sr content in wood and the degree of enrichment during combustion, the radionuclide values in the ash can become relevant for radiation protection [7].

In 1996, ashes of tree trunks (similar to chip and log ashes) from 18 sites in Upper Austria were investigated on behalf of the Upper Austrian provincial government [15]. The activity concentration of ^137^Cs was higher in the outer annual rings than in the heartwood. This is related to the better nutrient supply in the growth zones. The activity concentration of ^137^Cs in the different trees varied strongly. This variation again points to the site- and species-specific relationships in the transfer of ^137^Cs into the wood.

Nowadays, pellets are increasingly used, which behave differently from wood chips due to improved combustion systems. Considering the limited data available for Austria, the ongoing energy development trends, and the pursuit of a circular economy, a monitoring of radioactivity in wood fuels was carried out from 2020 to 2022 in Austria [25]. The hypothesis is that the results of the monitoring will confirm that there is no immediate danger to the population in Austria. Even for wood pellets, which have the lowest ash content compared to other wood fuels, it is expected that the accumulation of radioactive substances in the ash will not reach the reporting values in the EU.

## Materials and Methods

From 2020 to 2022, the Austrian Agency for Health and Food Safety (AGES) conducted a monitoring project [25]. The wood’s precise origin is often unclear, as wood pellet bags typically list various production sites, mainly different sawmills, without specifying the wood’s source. Wood in Austria frequently originates from areas surrounding the production sites but may be supplemented with wood from other locations due to shortages. Additionally, certain domestic companies operate sawmills both within Austria and abroad.

In this study, all 69 wood fuel samples were purchased in Austria or ordered online from Austrian providers. The production sites of the analysed samples are located in Austria, the USA, Czech Republic, Serbia, Romania, Latvia, and Ukraine. In total, 69 wood fuel [7 chips, 1 log, 58 pellets and 3 briquette (spruce wood)] and 27 wood ash [7 chips, 1 log, 15 pellets, 1 charred pellets (pellets ordered from the USA to smoke meat) and 3 briquette] samples were analysed.

Five wood chip samples are from private Austrian households with private forests in Austria and were logged by the consumers themselves. The chips are produced for the respective own use. The chips of another sample were bought by the consumer from a local farm with a private forest in Austria. Another chip sample is waste wood for a heating plant.

The log sample was brought to AGES by a concerned Austrian citizen after he bought it at a local hardware store and found out that the logs came from Ukraine.

Different pellets brands were purchased in stores. In total, 16 of the 58 wood pellet samples are from private Austrian households. With one exception (origin: USA; used to smoke meat) the pellets were produced in Europe. Most of the pellet samples were product samples, which were provided by the BEA Institute of Bioenergy and the Holzforschung Austria–Austrian Forest Products Research Society all under permission by proPellets Austria. Their exact production site is known but kept confidential (see Table 2).

**Table 2:** Raw data and sample description.

Sample type	Fuel sample	country of origin	^137^Cs activity concentration, Bq kg^−1^	Ash sample	^137^Cs activity concentration in ash, Bq kg^−1^	^90^Sr activity concentration in ash, Bq kg^−1^
Wood chips	C1	Austria	2.27 ± 0.24	CA1	194 ± 13	
	C2	Austria	<0.19	CA2	11.80 ± 0.87	
	C3	Austria	0.37 ± 0.14	CA3	36.8 ± 3.0	
	C4	Austria	5.22 ± 0.40	CA4	219 ± 15	531 ± 77
	C5	Austria	0.75 ± 0.15	CA5	18.4 ± 1.3	
	C6	Austria	1.02 ± 0.11	CA6	56.0 ± 3.9	
	C7	Austria	1.32 ± 0.17	CA7	118 ± 11	
Wood log	L1	Ukraine	0.327 ± 0.077	LA1	113.7 ± 9.0	
Wood pellets	P1	Austria	2.26 ± 0.37			
	P2	Austria	2.09 ± 0.35	PA2	413 ± 33	644 ± 93
	P3	Austria	1.18 ± 0.34	PA3	405 ± 32	465 ± 67
	P4	Austria	2.95 ± 0.32	PA4	398 ± 32	785 ± 81
	P5	Czechia	0.94 ± 0.26			
	P6	Austria	2.06 ± 0.34			
	P7	Czechia	1.67 ± 0.31			
	P8	Austria	2.44 ± 0.37			
	P9	Austria	1.11 ± 0.21			
	P10	Austria	1.88 ± 0.37			
	P11	Czechia	0.46 ± 0.18	PA11	267 ± 22	530 ± 77
	P12	Austria	2.53 ± 0.38	PA12	223 ± 18	
	P13	USA	<0.47	PC13	<0.80	
	P14	Austria	2.62 ± 0.37	PA14	338 ± 27	
	P15	Austria	2.77 ± 0.40	PA15	867 ± 68	690 ± 100
	P16	Confidential	0.65 ± 0.26			
	P17	Confidential	2.10 ± 0.28			
	P18	Confidential	0.69 ± 0.21			
	P19	Confidential	2.15 ± 0.36			
	P20	Confidential	1.15 ± 0.27			
	P21	Confidential	0.60 ± 0.25			
	P22	Confidential	3.59 ± 0.37			
	P23	Confidential	3.76 ± 0.46			
	P24	Confidential	0.92 ± 0.22			
	P25	Confidential	2.61 ± 0.39			
	P26	Confidential	2.47 ± 0.48			
	P27	Confidential	2.29 ± 0.35			
	P28	Confidential	1.79 ± 0.36			
	P29	Confidential	2.63 ± 0.36			
	P30	Confidential	5.39 ± 0.47			
	P31	Confidential	2.21 ± 0.36			
	P32	Confidential	2.68 ± 0.39			
	P33	Confidential	2.28 ± 0.36			
	P34	Confidential	1.51 ± 0.31			
	P35	Confidential	1.68 ± 0.34			
	P36	Confidential	1.04 ± 0.30			
	P37	Confidential	8.36 ± 0.78			
	P38	Confidential	1.17 ± 0.33			
	P39	Confidential	1.66 ± 0.43			
	P40	Confidential	1.72 ± 0.35			
	P41	Austria	2.74 ± 0.37	PA41	692 ± 54	560 ± 110
	P42	Austria	1.77 ± 0.25	PA42	356 ± 28	
	P43	Austria	1.23 ± 0.23	PA43	195 ± 16	
	P44	Austria	1.99 ± 0.34	PA44	390 ± 31	810 ± 120
	P45	Confidential	1.83 ± 0.35			
	P46	Confidential	3.10 ± 0.42			
	P47	Confidential	2.30 ± 0.35			
	P48	Confidential	1.12 ± 0.33			
	P49	Confidential	3.81 ± 0.44			
	P50	Confidential	2.19 ± 0.41			
	P51	Confidential	2.27 ± 0.37			
	P52	Confidential	1.26 ± 0.32			
	P53	Confidential	2.29 ± 0.35			
	P54	Austria	1.58 ± 0.15	PA54	372 ± 30	
	P55	Austria	2.93 ± 0.25	PA55	386 ± 31	532 ± 77
	P56	Austria	2.03 ± 0.38			
	P57	Austria	3.73 ± 0.52	PA57	759 ± 75	750 ± 110
	P58	Austria	1.90 ± 0.35	PA58	146 ± 15	
Wood briquettes with a hole	B1	Austria	3.03 ± 0.38	BA1	704 ± 55	1200 ± 180
	B2	Austria	3.97 ± 0.64	BA2	249 ± 25	363 ± 53
	B3	Austria	0.49 ± 0.38	BA3	129 ± 13	

The ash samples from burnt wood fuel were collected from 26 private households located throughout Austria, each using combustion systems of various brands and models. Additionally, a sample was obtained from one incineration plant that operates using wood chips.

For this study, the activity concentrations of wood ash handled by the public was of interest. No wood fuels were burnt in the laboratory, because the degree of radionuclide enrichment during combustion depends on various factors (e.g. heat curve, boiler shape) which can not exactly be recreated in the laboratory.

The nomenclature for the different samples can be taken from Table 2.

### Cesium analysis

The ^134^Cs and ^137^Cs activity concentration in wood fuels and their ashes was determined by the AGES accredited measurement laboratory using gamma-ray spectrometers with high-purity germanium detectors according to ISO 20042:2019 [26]. The spectra were analysed using the Genie2000 spectra analysis program (version 3.4.0).

The pellet (non-destructive method) and crushed briquette samples were measured in standard 250 ml beakers, while the chip and hackled log samples were measured in standard 3-liter Marinelli beakers with predefined 2-liter geometry. The ash samples were compacted into suitable geometries. The variety of the samples were measured for at least 8 hours.

### Strontium analysis

^90^Sr analyses were performed after chemical separation using a PerkinElmer 1220 Quantulus™ liquid scintillation counter by the AGES accredited measurement laboratory according to ISO 18589-5:2019 [27].

In preparation of the chemical separation, 10 g of the wood ash has been ashed at 200°C for 180 min, at 500°C for 240 min and followed by 300 min at 700 °C to complete the incineration process.

Assisted by microwave, the ashes have been digested using 12 ml conc. HNO_3_ s.p and 2 ml H_2_O_2_ 30% at 180°C for 90 min. The sample was filtered through a blue ribbon filter.

To estimate the concentration of natural strontium in the sample, an aliquot of the digested sample was taken for ICP-MS measurement. 0.2 ml of a strontium carrier solution (5 mg ml^−1^) has been added afterwards to the sample. The sample was evaporated nearly to dryness and dissolved in 30 ml of 8 M HNO_3_. To determine any loss of strontium in this first step, two aliquots have been taken for ICP-MS measurement of natural strontium. One drop of n-octanol has been added to saturate the loading solution.

For the chemical separation, in 40 ml Milli-Q saturated with n-octanol 5.53 g Sr® resins put into a 10 cm column. After preconditioning the column with 8 M HNO_3_ the sample solution has been loaded, the time of chemical separation has been noted. The column was washed with 70 ml of 8 M HNO3/n-octanol to elute Yttrium. After further washing steps (10 ml of 3 M HNO_3_–0.05 M oxalic acid/n-octanol, 10 ml of 3 M HNO_3_/n-octanol) strontium was eluted with 60 ml of 0.05 M HNO_3_, while the first 10 ml of the elution has been discarded. The strontium eluate was evaporated nearly to dryness and was dissolved in 8.12 ml of 0.05 M HNO_3_ and transferred to an LSC-vial. Two aliquots of 0.06 ml were taken to determine the chemical recovery by measuring natural strontium content. In total, 12 ml of the OptiPhase HiSafe 3™ by PerkinElmer was added to the LSC-vial and measured for 6 cycles á 80 min by liquid scintillation counter 2 weeks after separation according to ISO 18589-5 [27].

## Results and Discussion

**Figure 1:**
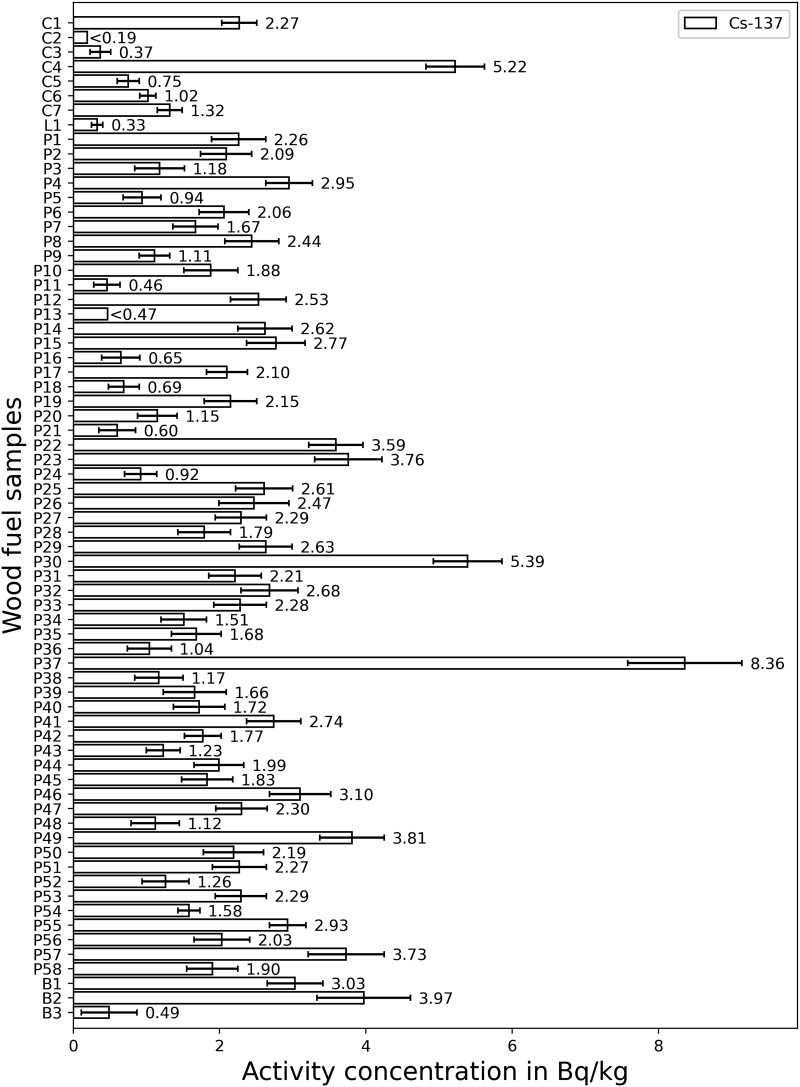
Measured activity concentration of ^137^Cs in wood chips (C), logs (L), pellets (P) and briquettes (B). Sample numbers see Table 2.

**Figure 2:**
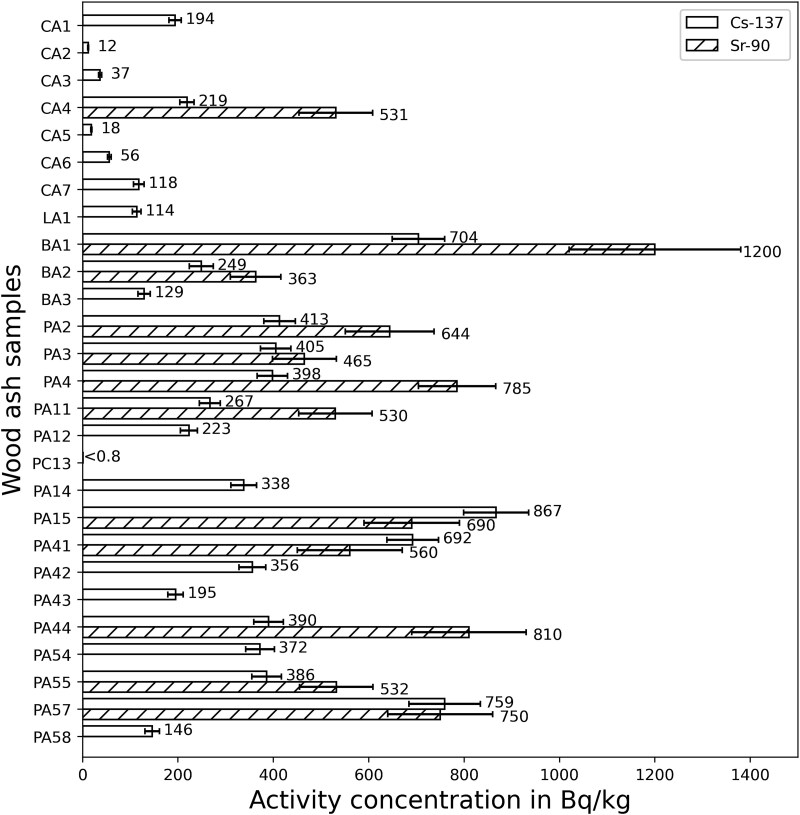
Measured activity concentration of ^137^Cs and ^90^Sr in wood ash of chips (CA), logs (LA), pellets (PA, PC are the charred pellets) and briquettes (BA). Sample numbers see Table 2 (‘A’ in the numbering indicates an ash sample).

The results for the activity concentration of ^137^Cs in wood fuels are shown in Fig. 1. The results for the activity concentration of ^137^Cs and of ^90^Sr in wood ash are shown in Fig. 2. The raw results for the different samples can be taken from Table 2.

### Activity concentration in wood fuels

The wood fuel samples were not dried especially, but water content of the pellets and briquettes is standardized and was therefore always less than 10 wt%. The water content of the chip and log samples was not determined. However, the samples were stored for some time before analyses and therefore water contents of less than 20 wt% can be expected.

#### ^134^Cs results in wood fuels

The activity concentration of ^134^Cs was below the detection limit (<0.64 Bq kg^−1^) for all samples.

#### ^137^Cs results in wood chips and logs

In this study, the ^137^Cs activity concentration of one wood chip sample (C2) from a forest in Lower Austrian was lower than the detection limit of 0.19 Bq kg^−1^. The minimum value found in chips is (0.37 ± 0.14) Bq kg^−1^ (sample C3), while the maximum value is given by (5.22 ± 0.40) Bq kg^−1^ (sample C4).

The log sample (L1) originating from the Ukraine had a ^137^Cs activity concentration of (0.327 ± 0.077) Bq kg^−1^.

The average ^137^Cs activity concentration of the chips and the log is 1.61 Bq kg^−1^ (The measurement below the detection limit was not considered for the calculation.).

The results of this study for wood chips and logs are lower than in other publications [10], where e.g. the national maximum ^137^Cs activity concentration in Lithuanian chips was (28 ± 7) Bq kg^−1^ and of all samples the maximum activity concentration value of ^137^Cs (32 ± 2) Bq kg^−1^ was provided by a chip sample from Belarus.

Generally, the consumers of the wood fuels do not know which tree species are used. However, the significance of tree species for the accumulation of radionuclides was demonstrated in relation to the ^137^Cs activity concentration, with values reaching 1.3 Bq kg^−1^ in oak, 11.0 Bq kg^−1^ in beech wood, and 8.2 Bq kg^−1^ in sycamore wood [14].

#### ^137^Cs results in wood pellets and briquettes

The lowest measured ^137^Cs activity concentration in wood pellets is from the Czech Republic with (0.46 ± 0.18) Bq kg^−1^ (sample P11). The highest measured ^137^Cs activity concentration in pellets in this study, of (8.36 ± 0.78) Bq kg^−1^ (Sample P37 originates from the same country (confidential) as P38, P39, and P40, with ^137^Cs activity concentration between 1.17 and 1.72 Bq kg^−1^.), closely aligns with the highest ^137^Cs activity concentration found in pellets from ten different dealers in 2009, as reported by the German Energy Pellet Association (DEPV), which was 7 Bq kg^−1^ [16]. However, it is approximately double the highest recorded value of 4.6 Bq kg^−1^ reported by proPellet Austria in 2009 from production sites in the Czech Republic, Germany, and Austria [16].

The average of ^137^Cs in this study lies by 2.18 Bq kg^−1^ (The measurement below the detection limit was not considered for the calculation.), which is in good agreement with the average of ^137^Cs activity concentration of 2.93 Bq kg^−1^ (22 wood pellet samples) reported by Desideri *et al.* [28]. Ladygienė *et al.* [10] reported that the highest ^137^Cs activity concentration in Lithuanian pellets was (14 ± 1) Bq kg^−1^. Imported darker pellets (They are made from wood with bark and are therefore cheaper.) from the Ukraine to Lithuania had a maximum of (156 ± 9) Bq kg^−1^ for dry weight. The highest reported ^137^Cs activity concentration of 65 pellet samples (average 13.2 Bq kg^−1^) gathered from 2010 to 2012 was an Ukrainian sample with about 130 Bq kg^−1^ [11].

3 different wood briquettes were analysed and showed an average of the ^137^Cs activity concentration of 2.50 Bq kg^−1^ ranging between (0.49 ± 0.38) (sample B3) and (3.97 ± 0.64) Bq kg^−1^ (sample B2), which is in good agreement with the reported maximum ^137^Cs activity concentration of (1.4 ± 0.4) Bq kg^−1^ for Lithuanian briquettes [10].

The activity concentrations of ^137^Cs in wood fuels available in Austria are in good agreement with the European literature and confirm there is currently no health risk for the population from wood and wood fuels itself, since they are harmless from the point of view of radiation protection. All measured activity concentrations of ^137^Cs are below the limit values or reporting values given in the introduction. The lowest limit value found for the activity concentration of ^137^Cs is 10 Bq kg^−1^ in dry wood and applies to wood imported to Latvia [19].

### Activity concentration in the wood ashes

The disposal of ashes in private households is regulated differently in each federal state and often also in each municipality. In most parts of Austria, it is compulsory to dispose of cooled ashes in the residual waste, which is designated for non-recyclable items such as sanitary products, heavily soiled packaging and non-recyclable plastics. The heating plant deposits the produced ash at a landfill site. In total, 14 private households mainly reuse the produced wood ashes (5 chip ashes, 1 log ash, 7 pellet ashes, and 1 briquette ash) in their gardens (e.g. compost heap). The predominant reason cited by residents for reusing wood ash in their gardens is a long-standing tradition of using it as a fertilizer. Two of these 14 households (1 chip ash and 1 briquette ash) sometimes and 10 private households (7 pellet ashes, 1 charred pellets, and 2 briquette ashes) always dispose the ash in the residual waste. Two households did not specify their disposal pathway of their ashes.

#### ^134^Cs results in wood ashes

The activity concentration of ^134^Cs was below the detection limit (<8.1 Bq kg^−1^) for all samples.

#### ^137^Cs results in wood chip and log ashes

The ^137^Cs activity concentration of the wood chip ash ranged between (11.80 ± 0.87) (sample CA2) and (219 ± 15) Bq kg^−1^ (sample CA4) with an average of 96 Bq kg^−1^.

The measured ^137^Cs activity concentration in this study are significant lower than previously reported maximum values of (7201 ± 567) Bq kg^−1^ (origin: Lithuania) and (2626 ± 118) Bq kg^−1^ (origin: Belarus) [10].

Nevertheless, the results of this study are in good agreement with the observed ^137^Cs activity concentrations in ashes from Bulgarian and Romanian wood, which ranged from 31 to 64 Bq kg^−1^, and with ashes from Greek wood, which had values ranging from 25 to 499 Bq kg^−1^ [14].

#### ^90^Sr results in wood chip ash

The ^90^Sr activity concentration was only analysed in one wood chip ash sample due to their higher ash content compared to pellets and briquettes. Therefore, the accumulation of mineral salts, heavy metals and radionuclides in chip and log ash is in general lower than for the other wood fuel ashes. This means less possible radiological risk from chip and log ash compared to pellet and briquette ash.

In this study, the analysed ^90^Sr activity concentration of chip ash is (531 ± 77) Bq kg^−1^ (sample CA4). The sample also has the highest ^137^Cs activity concentration (wood type: spruce).

The highest recorded domestic activity concentration of ^90^Sr in Lithuania was (1508 ± 230) Bq kg^−1^ in ash from Lithuanian chips, while the highest foreign value was obtained from a chip sample from Belarus, measuring (634 ± 98) Bq kg^−1^ [10].

#### ^137^Cs results in wood pellet and briquette ashes

The charged wood pellet sample (PC13), which was used to smoke meat, had a ^137^Cs activity concentration below the detection limit. The lowest ^137^Cs activity concentration of the 15 pellet ash samples was (146 ± 15) Bq kg^−1^ (sample PA58) and the highest (867 ± 68) Bq kg^−1^ (sample PA15). The average of ^137^Cs is 414 Bq kg^−1^ (The measurement below the detection limit was not considered for the calculation.), which is comparable to the average of 571 Bq kg^−1^ from Desideri *et al.* [28].

Significantly higher values were reported by Ladygienė *et al.* [10], where the domestic maximum value for the ^137^Cs activity concentration was (2630 ± 121) Bq kg^−1^ in ash from Lithuanian pellets, and the maximum value for the ^137^Cs activity concentration (9800 ± 700) Bq kg^−1^ was recorded in a pellet ash sample from Ukraine.

The ^137^Cs activity concentration in wood briquette ash ranges from (129 ± 13) (sample BA3) to a maximum of (704 ± 55) Bq kg^−1^ (sample BA1) (arithmetic mean 361 Bq kg^−1^), which is half the reported maximum in Lithuanian briquette ash (1489 ± 95) Bq kg^−1^ and more than eight times lower than the published maximum of (5754 ± 485) Bq kg^−1^ in a Ukrainian sample [10].

#### ^90^Sr results in wood pellet and briquette ashes

Nine of the 15 wood pellet ash samples were analysed for ^90^Sr. The activity concentration ranged between (465 ± 67) (sample PA3) and (810 ± 120) Bq kg^−1^ (sample PA44) (the arithmetic mean resulted to 641 Bq kg^−1^). The results of this study align well with a dataset of 22 pellet ash samples, which yielded a mean ^90^Sr activity concentration of 685 Bq kg^−1^ [28] as well as with the 2010 study by Ladygienė *et al.* [10], where the highest ^90^Sr activity concentration for Lithuanian pellet ash was (469 ± 73) Bq kg^−1^, while the overall maximum of (715 ± 107) Bq kg^−1^ was recorded in ash from a pellet sample from Ukraine.

Two of the 3 wood briquette ash samples were analysed for ^90^Sr. The activity concentration found were (363 ± 53) (sample BA2) and (1200 ± 180) Bq kg^−1^ (sample BA1). The arithmetic mean of 782 Bq kg^−1^ in this study closely matches the reported maximum ^90^Sr activity concentration at (410 ± 63) Bq kg^−1^ in a briquette ash sample from Ukraine and the maximum ^90^Sr activity concentration of (712 ± 106) Bq kg^−1^ in ash from Lithuanian briquettes [10].

### Comparison of results to other studies

No correlation was found between the activity concentration of ^137^Cs in the wood fuels or their ashes and the activity concentration of ^90^Sr in the ashes. As already mentioned, the activity concentrations vary greatly depending on the tree species, origin, and type of wood. The Handbook of Parameter Values for the Prediction of Radionuclide Transfer in Terrestrial and Freshwater Environments (2010) by the International Atomic Energy Agency provides different transfer factors for ^137^Cs and ^90^Sr for forest trees (^137^Cs: spruce, fir tree, pine, oak, beech, birch, and willow; ^90^Sr: alder, fir tree, pine, oak, aspen, and birch) [29]. Strontium is readily available to plants in the soil and only relatively small proportions of the total content are not exchangeable [30]. Artificial strontium follows the metabolic pathway of natural calcium, and uptake can be reduced by high calcium availability [30]. The tree can only absorb as much ^137^Cs and ^90^Sr as is present in the soil. The content in the soil dates back to the atmospheric nuclear weapons tests in the 1950s and 1960s and the reactor accident at Chornobyl in 1986. The extent of this radioactive fallout varied greatly from region to region. For ^137^Cs, there is precise documentation of the contamination of soils in Austria [31]. Therefore, if the origin and tree species of wood are known and for example, a heating power plant uses only this specific wood, it would be possible to determine a nuclide vector for ^90^Sr in the ashes by using the activity concentration of ^137^Cs of the wood itself or of the ashes, and this should be investigated in detail in the next project.

The results of the activity concentrations of ^137^Cs and ^90^Sr are in the range of other studies in Europe, and especially the pellet and briquette ashes could be relevant from a radiation protection point of view. The ash content is lower than for wood chips and logs. Therefore, the accumulation of mineral salts, heavy metals and radionuclides in wood pellet and briquette ashes is in general above than for the other wood chip and log ashes. The measured ^137^Cs activity concentrations are below 1000 Bq kg^−1^ for all ash samples, which is the limit value in Latvia, Belgium, and Italy. In Sweden, for the four ash samples (PA15, PA41, PA57, and BA1) with ^137^Cs activity concentrations above than 500 Bq kg^−1^, the ashes can be scattered in forest areas, but not on agricultural land.

Private households producing pellet or briquette ash were informed that they should dispose of the resulting ash with their residual waste. According to wood experts, consumers of wood pellets are already advised against scattering wood pellet ashes in their gardens or even on a vegetable patch [32, 33]. In addition to radionuclides, non-combustible minerals, salts, and heavy metals also accumulate in the ashes. In this study, the accumulation of radionuclides in ash is in average 3 times above than in chip ash (see Table 3). Due to the small sample size, the log sample was not taken into account.

**Table 3:** Accumulation of ^137^Cs from wood into the ash for the different wood fuel types.

Fuel type	Average ^137^Cs activity concentration in wood, Bq kg^−1^	Number of samples	Average ^137^Cs activity concentration in ash, Bq kg^−1^	Number of samples	Accumulation
Chips	1.6 ± 1.3	7	93 ± 72	7	58
Logs	0.327 ± 0.077	1	113.7 ± 9.0	1	348
Pellets	2.15 ± 0.81	58	390 ± 160	16	181
Briquettes	2.5 ± 1.4	3	360 ± 230	3	144

## Conclusion and Outlook

The measurements of ^137^Cs activity concentration of the wood fuels range between (0.327 ± 0.077) and (8.36 ± 0.78) Bq kg^−1^, which indicated no necessity for special regulations on wood imported to Austria. All measured ^137^Cs activity concentrations are below the limit of 10 Bq kg^−1^ for wood imported into Latvia, which is the lowest limit found for wood fuels [19].

The ^137^Cs activity concentration in the wood ash samples ranges between (11.80 ± 0.87) and (867 ± 68) Bq kg^−1^ and the ^90^Sr activity concentration ranges between (363 ± 53) and (1200 ± 180) Bq kg^−1^. The accumulation of radionuclides in ash depends on the tree species [9, 29], type of wood [9, 10, 14, 15] and the burned wood fuel. A single-family house in Austria, which uses wood fuels for room and water heating, produces around 30 kg wood ash per year. The exact amount of produced ash depends on the used kilowatt-hour, the type of wood and incineration plant. Most members of the public in Austria (14 out of 26 private Austrian households) use wood ash in their private gardens.

A dose assessment for the use of wood ash as a fertilizer in private gardens will be carried out in a separate study. Dose assessment is a crucial process in radiation protection that involves determining the amount of radiation to which a person or population has been exposed. The assessment of radiation dose will help to evaluate potential health risks better to ensure that radiation exposure remains within safe limits.
